# Strategic European partnerships for UK universities post-Brexit: navigating a globally contested field of world-class universities

**DOI:** 10.1007/s11233-023-09123-0

**Published:** 2023-05-12

**Authors:** Ludovic Highman, Simon Marginson, Vassiliki Papatsiba

**Affiliations:** 1 School of Management, University of Bath, Bath, UK; 2 Department of Education, University of Oxford, Oxford, UK; 3 School of Social Sciences, Cardiff University, Wales, UK

**Keywords:** Field, Higher education, Bourdieu, Comprehensive strategic partnerships, Position-takings, Internationalisation

## Abstract

This paper assesses how UK universities seek to maintain their global dominant position post-Brexit through comprehensive strategic partnerships with key European institutions as part of their internationalisation strategies. Drawing on 24 semi-structured interviews conducted from November 2017 to July 2018 in 12 UK universities vertically differentiated and spread along the highly hierarchised spectrum of British universities in all four nations, we aim to examine which types of universities are most inclined to form international comprehensive university-wide strategic partnerships, and how they identify their partners. The analysis is framed within Bourdieu’s theory of “economy of practices” which considers all university practices as economic practices that are ultimately tailored towards maximising either material or symbolic profit. Unlike in business and industry, where organisations traditionally compete to maximise profit, universities must both compete and collaborate with one another in order to improve (or maintain) their position in the field. UK universities will need to navigate the post-Brexit space they find themselves thrown into, and in the process will need to review international institutional links with both European Union (EU) based and non-EU universities. This paper will assess how UK universities seek to maintain their dominant position in the field through comprehensive strategic partnerships with key foreign institutions.

## Introduction

Global challenges such as climate change, gender equality, fighting poverty and pandemics necessitate cross-border and cross-disciplinary collaboration. Equally pressing is the advancement of cutting-edge scientific knowledge that occurs in a context of international research collaboration and authoritative collective judgement. The latter is, by nature, not confined to national borders. Marginson (2008) argues that global higher education is not a level playing field. It is dominated by English-speaking countries with reputable higher education systems and institutions, whose dominance is further catalysed through research, where “a single mainstream system of English-language publication of research knowledge” (p. 303) marginalises other research outputs. Global higher education is also affected by how institutions position themselves in three popular (Elken et al., 2016) global university rankings (Academic Ranking of World Universities [ARWU], Quacquarelli Symonds [QS], Times Higher Education [THE]). In the United Kingdom (UK) higher education system(s), which provided the national context for this research, institutions have over time organised themselves into membership groups reflecting their different missions. UK universities are vertically differentiated (Boliver, 2015; Shattock, 2017), with institutions grouped into membership organisations that situate them along an imaginary hierarchy where research intensity is the principal determinant of prestige. These hierarchies, consisting of relations of dominance and subordination between actors with different degrees of authority, are often inflected and shaped by national contexts. In the UK, status differentiation can be the result of research activity, the quality of the teaching and learning experience, economic resources at the disposal of an institution, academic selectivity and the socioeconomic mix of students enrolled (Boliver, 2015) while different structural models of higher education have implications in terms of regulatory and funding frameworks different types of higher education institutions may find themselves aligned to (Kyvik, 2004). The extent to which their domestic status directly maps onto parallel global hierarchies requires closer examination.

Post-Brexit, UK universities have made it clear they wish to remain internationally connected and global in their outlook (Russell Group, 2021; University of Oxford, n.d.) which is unsurprising since nine out of ten academics supported the UK remaining in the European Union [EU] (Finn, 2021). This objective is challenging because of the loss of access to the Erasmus + scheme confirmed late 2020. Erasmus + encompasses activities including student and trainee mobility, staff mobility, vocational education and training, projects for youth, sport actions and learning mobility activities for school pupils and staff (Hubble et al., 2021). Between 1987 and the UK’s withdrawal, 200,000 UK based individuals studied and worked in Europe (Adams, 2020). Furthermore, the sector has suffered years of uncertainty as to whether the UK would associate to Horizon Europe, the EU’s largest ever multiannual framework programme for research and innovation. Remaining internationally connected must therefore also be secured outside high-level politically negotiated frameworks through institution-led partnerships with foreign counterparts, in particular in Europe, where loss of access to reciprocal mobility schemes and potential loss of research income and access to research partnerships are realities UK universities must navigate, through initiatives of their own (Highman, 2019).

While there is a seemingly non-exhaustive list of potential partners, the reputational “iron cage” (DiMaggio & Powell, 1983) has a strong grasp on the minds of those working in higher education. For many institutions, a strategic partner must reflect or add value to the profile, standing and reputation of their own institution. This article aims to assess which types of universities appear to be best prepared to enter comprehensive university-wide strategic partnerships with foreign universities, how and why they identify partners. To do so, this article aims to unearth the underlying rationale(s) for selecting strategic partners for cross-border institutional collaboration in Europe post-Brexit and how this determines the type of institution that is most likely to focus on this category of inter-institutional partnership, rather than other forms of internationalisation encountered under the increasing transnationalisation of UK universities (Kleibert, 2023) and that have existed before Brexit (Altbach, 2015; Havergal, 2018; Healey 2013; Healey, 2015; Wilkins, 2016). In other words, how and why are certain partners chosen by UK universities when selecting European partners and does this further contribute to structure a global field of higher education, with agents (i.e., universities) positioned and position-taking within the field (Bourdieu, 1993; Marginson, 2008)? The analysis is framed within Bourdieu’s “general science of the economy of practices” (1993, p.8) that enables one to analyse all university practices (that originate in agents’ learned dispositions) as economic practices ultimately tailored towards maximising either material or symbolic profit.

Internationalisation has been defined as “the process of integrating an international dimension into the major functions of a university or college” (Knight, 1999, p.2) implying an institution-wide scope that integrates such a dimension into all core missions of a university. Elkin, Farnsworth and Templer (2008) offer a breakdown of various dimensions often understood to constitute internationalisation, of which one is of particular importance to this study: “international institutional links” (p.243). Such links require cross-border partners to work closely and on a multitude of areas encompassing their teaching, research and service missions, with an assumption that their activities and missions overlap. We argue that international institutional linkages have become an umbrella dimension that incorporates internationalisation activities such as student and staff mobility, staff interaction in an international context, internationally focused programmes of study, and cross-border research collaboration. It potentially includes attracting postgraduate international students who completed their undergraduate degree at a partner institution through various financial support schemes. These institution-wide links may not always overlap with the diversity of partnerships individual academics pursue as there is an inherent tension between an institutional strategy and individual choices.

This concentration and streamlining of internationalisation activities under carefully selected international institutional links is core to understanding the motivations of UK universities when engaging in Europe, a region that is presumably less affected by the legacies of international education that continue to shape modern neo-colonial relations of education between the UK and other regions of the world (Madge et al., 2009) and where asymmetric power relations in the global education field are not as exacerbated as in post-colonial contexts (Siltaoja et al., 2019; Le-Ha, 2017; Leung & Waters, 2017). International institutional links are therefore viewed as an all-encompassing dimension that sometimes precedes and other times trails other internationalisation activities. It is in this sense that the article will refer to international institutional links as comprehensive strategic partnerships encompassing different activities and we argue that some UK universities are seeking to merge many of their internationalisation activities under carefully selected linkages with Europe.

This empirical qualitative study is based on research conducted in 12 UK universities, selected to be as representative as possible, including universities from different membership groups, age, research intensity, location in Remain and Leave voting constituencies, and geographical distribution. Following a discussion of the theoretical framework underpinning this article based on Bourdieu’s social field of higher education and the inherent power struggle between agents structuring this field, with institutions competing for resources and recognition, the findings will be presented alongside the “different organizational tiers” (Friedman, 2018, p.438) that are characteristic of the structure of British higher education. The discussion will focus on the priorities driving interinstitutional international collaboration, through the perspectives of senior professional and academic management, depending on institution type and position within the field, and how differentiated institutional characteristics contribute to further structuring a global field of higher education.

## Theoretical framework: towards a defined social (sub)field of global higher education?

According to Bourdieu (1993), social formations are organised around a complex ensemble of social fields in which various forms of power circulate and in which agents internal to the field struggle for control over resources, although not always based on conscious calculation. Each field is conceptualised as possessing a high degree of autonomy and is “defined as a structured space with its own laws of functioning and its own relations of force independent of those of politics and the economy, except, obviously, in the cases of the economic and political fields” (Bourdieu, 1993, p.6).

Bourdieu (1993) argues that a field (i.e., the literary or artistic field) is structured by an inherent opposition between on the one hand what Marginson has referred to as an “elite subfield of restricted production” (2008, p.305), and on the other a “subfield of large-scale mass production” (ibid.) favouring commercial imperatives. Within each subfield, different principles of hierarchisation operate, with on the one hand a subordinate and heteronomous subfield more easily subjected to external controls and impositions and on the other a dominant and autonomous subfield, and therefore least responsive to external demands involving economic capital and market demands. Bourdieu’s polarity and the principles of hierarchisation operating within opposing poles, or “sub-fields” (Bourdieu 1993 , p.46) can be transposed to the field of higher education and explain relations of power within national and global systems. Positions in the field depend on the amount of material and non-material resources an agent possesses in relation to other occupants. He refers to these resources as capital.

Academic capital is crucial to our understanding of who UK universities decide to associate themselves with, through international institutional linkages. Bourdieu (1988) refers to academic capital in previous work (Kloot, 2009; Naidoo, 2004) as manifested through the power to hold a position enabling domination of other positions and their recipients. Such academic capital is closely related to position within the institutional hierarchy, and ultimately control over the instruments of reproduction of the university body, as opposed to intellectual or scientific capital derived from a scholarly reputation or intellectual renown (Bourdieu, 1988; Naidoo, 2004). However, in later work (Bourdieu, 1996), academic capital has been defined as an “institutionalized form of cultural capital based on properties such as prior educational achievement, a ‘disposition’ to be academic (seen, for example, in manner of speech and writing), and specifically designated competencies” (Naidoo, 2004, p.458). Our use of the term academic capital is to be understood in the latter sense, and we will combine it with “scientific capital” (Bourdieu, 1988, p.99), which can be measured through the consecration accorded by the scientific field, particularly overseas, through publications, citations and translations (Kloot, 2009).

To further operationalise these concepts, an institution’s academic capital can be understood at a holistic level, by virtue of the academic capital held by its students and staff, while its scientific capital refers to the intellectual renown of its academics, best illustrated through research achievements. Both types of capital are therefore embodied in the teaching and research missions of a university. International institutional linkages between universities that cover teaching and research activities can be conceptualised as a gateway for exchange of capital and would presumably involve partners to provide evidence of sufficient capital. This may favour a certain type of institution, namely the more research focused institution, in developing such links, because of the requirements of and benefits associated with international research collaboration (Adams & Gurney, 2018). Based on this, one might expect a propensity for research universities to seek such international institutional linkages because of their comprehensive nature and the ability to articulate many internationalisation activities through these institution-wide linkages. This is particularly relevant in a Brexit context, which represents an exogenous shock which has disrupted intra-regional student mobility flows towards the UK (Fazackerley, 2020) and EU research funding opportunities for UK universities (Fazackerley, 2023) with potential implications with regard to their ‘partnerships politics’.

The institutional academic capital can be illustrated by academic staff qualifications, graduation from an elite institution, or average entry tariff of the student body, a direct result of the institution’s capacity to draw on its symbolic power to set specific recruitment policies and entrance requirements. Furthermore, drawing on the work of Bourdieu’s habitus (1993), which is an acquired set of beliefs and capabilities which leads an agent to act or think a certain way, not always consciously, the concept of “institutional habitus” (Reay et al., 2001) develops the idea in relation to higher education institutions. The institutional habitus can thus be understood to refer to more than the culture of the organisation, and include relational issues and priorities, “which are deeply embedded, and sub-consciously informing practice” (Thomas, 2002, p.431). The concept is useful to understand agents, in the sense of higher education institutions, as having developed a distinct institutional habitus, and how in turn an institution’s values, practises and mission(s) are reflected in their comprehensive strategic partnerships such as international institutional linkages.

This article will argue that because of the specific interests of the field, one of the strategies of accumulation utilised by the agents involved is to identify other dominant players with similar holdings of academic and scientific capital to collaborate with, through a principle of equivalency aimed first and foremost at guaranteeing stability and position within the field. This is particularly relevant in the context of severing ties with Europe and considering that Europe is a region comprising highly industrialised states with quality education systems and leading universities that make many types of inter-institutional partnerships less appealing to host nations and universities. The concepts of capital, field and habitus will be applied to explore the interdependency between the prior positions of institutions and the position-taking strategies they select. The data indicates that universities positioned within the subfield of elite universities have a disposition to enter into bilateral partnerships and multilateral alliances with institutions they identify as occupying symmetrical dominant positions or where possible partnering up with an institution perceived to be of higher status. The latter will be the focus of this article, which aims to demonstrate the extent to which a subfield of elite universities sharing common characteristics seek to maintain their dominant position in a global (sub)field of elite universities through their internationalisation activities.

## Methodology

### Case selection and participants

The empirical basis of the investigation was a case study of 12 UK universities distributed across the four nations. Although the objective was to have a total of 12 case study universities, 20 had to be contacted in order to find 12 willing to participate. In this paper the drivers and rationale for deciding upon international institutional links are analysed, based on qualitative data extracted from 24 semi-structured interviews. All interviews were voice-recorded, then transcribed, coded and analysed manually and using NVivo. The data were collected in a context permeated by Brexit-related uncertainty following the results of the June 2016 Brexit referendum, between November 2017 and July 2018.

The sample includes two London-based institutions (including one specialised institution), two Scottish universities, one Welsh university, one institution in Northern Ireland and six English, universities located in the Midlands, the North and the South West. The sample was geographically widespread and covered various institution types from large research-intensive (i.e., members of the Russell Group with a very high research activity and over 18,000 enrolled students), smaller research-led institutions (i.e., institutions with an enrolment figure inferior to 15,000 students and not members of the Russell Group but with a significant research output, demonstrated by numbers of publications belonging to the top 10% most frequently cited – but with less than 3,000 publications overall and less than 400 publications for the 2014–2017 period belonging to the top 10% most frequently cited as recorded by the Centre for Science and Technology Studies [CWTS] [Leiden University, 2021]) to more teaching-focused institutions, a heterogenous category including former polytechnics still referred to as “post-1992 universities” (Boliver, 2015, p.609; Deem & Brehony 2005, p.224) and an institution in Northern Ireland that is the result of a cross-binary merger between a university and a polytechnic that predates the 1992 Further and Higher Education Act.

Boliver (2015) uses cluster analysis to establish four university groupings based on research quantity and quality, student satisfaction with teaching, endowment and investment income, spending on academic services per student, student-staff ratios, academic and social selectivity of student intake, and outcomes for degree holders. The aim was to cover the clusters of UK universities identified in the literature, although this was not possible because many institutions refused to participate.

**Table 1:** Sample of case study institutions and clusters they belong to (Boliver, 2015)

Research-intensive universities	Research-led universities	Teaching-focused universities
(Russell Group members with > 18,000 students registered in 2016-17 and > 3,000 publications for 2014-17 and > 400 publications belonging to the top 10% most frequently cited in their field)	(Institutions with < 15,000 students registered in 2016-17 and with < 3,000 publications for 2014-17, and < 400 publications belonging to the top 10% most frequently cited in their field)	
London Russell Group university (cluster 2)	London specialised university (cluster 2)	Midlands post-1992 university (cluster 3)
Northern Russell Group university (cluster 2)	Midlands research-led university (cluster 3)	Northern post-1992 university (cluster 3)
		Northern Irish institution (cluster 3)
North East Russell Group university (cluster 2)	Scottish research-led university 1 (cluster 2)	Post-1992 Welsh university * (clusters 3 and 4)
South West Russell Group university (cluster 2)	Scottish research-led university 2 (cluster 2)	

* This institution is the result of a merger between former institutions categorised under clusters 3 and 4 (Boliver, 2015)

In each institution, two senior staff members involved in internationalisation activities (e.g., cross-border research collaboration, student and staff recruitment, student exchange, institutional strategy planning) were interviewed. In ten out of 12 institutions the sample included one participant from an academic background coupled with a senior management role (e.g., Pro Vice-Chancellor) and one non-academic participant in a professional senior role with direct involvement in internationalisation and/or strategic planning (e.g., Director of Global Engagement). In the remaining two institutions, because of staff availability and suitability for participation in the study, interviewees include two senior staff from an academic background who were in a senior managerial role either contributing to or overseeing the internationalisation activities of the institution. 24 participants were included in total (n = 24).

**Table 2:** Participants in each case study institution

Institution	Role of participant
Northern Russell Group university *	Associate Vice President 1 Associate Vice President 2
Northern post-1992 university	Pro Vice-Chancellor Director
North East Russell Group university	Pro Vice-Chancellor Director
South West Russell Group university	Senior executive leader Director
London Russell Group university	Vice-Provost 1 Vice-Provost 2
London specialised university	Pro-Director Director
Midlands research-led university *	Pro Vice-Chancellor Dean
Midlands post-1992 university	Deputy Vice-Chancellor Director
Northern Irish institution	Pro Vice-Chancellor Pro-Chancellor
Post-1992 Welsh university	Senior executive leader Senior corporate executive
Scottish research-led university 1	Senior Vice-Principal Director
Scottish research-led university 2	Senior Vice-Principal Director

* Within these universities, two academics both with senior executive positions were interviewed, in roles with close involvement or oversight of internationalisation activities (e.g., research)

### Limitations

Eight universities denied the research group access, either because of the alleged sensitivity created by the political situation or because of limited human resources. Research intensive, research-led and teaching-focused institutions declined to participate, including institutions categorised by Boliver in clusters 1, 2 and 3 (2015). The authors recommend further research encompassing more institutions, in particular from cluster 1, once there is more certainty in the sector. However, considering the politicised nature of the period in which the research took place, a group of 12 universities was deemed sufficient. A key limitation of this study is its focus on a phenomenon in flux and the temporality of the qualitative data collected in a fast-changing policy environment. Because of this, the study seeks primarily to shed light on the priorities that emerged in the aftermath of Brexit, and how universities sought to remain engaged with their European peers.

Although there was a risk that senior participants may be wary of divulging strategic matters, this proved to be unfounded, as most participants were keen to share the orientation of their institution’s internationalisation strategy. This area was less sensitive than one directly related to an institution’s financial sustainability. It was decided to focus on university staff at the senior level because in the UK, members of the senior management team are generally responsible for international institution-wide links, because of the strategic importance attached to such partnerships, and would be closely involved. By selecting a senior staff member from professional services and one with an academic background where possible (in ten out of 12 institutions), the authors attempted to ensure consistency and veracity by including both management and academic perspectives on the matter. Intra-institutional bargaining processes between senior management, middle management and frontline staff is not within the scope of this paper.

### Procedure

Purposive sampling was used to recruit participants for semi-structured interviews. During the interview, participants were invited to share their perceptions of the importance of internationalisation for their institution and their institutional approaches to inter-institutional international partnerships post Brexit. Interviews lasted between 45 minutes and one hour, were audio-recorded digitally and professionally transcribed. The semi-structured interview included questions such as “What is the current approach of your University with regard to European collaboration, in a general sense?”, “In terms of European collaboration, what are the priorities of your institution?”, “When you think European engagement or collaboration, what comes first in your mind”, “When you do engage with European universities, how do you engage with them, is this done through formal agreements or rather through informal research networks”, “How do you think your University will interact with other EU universities post-Brexit?”.

### Data analysis

Through a careful review of 24 interview transcripts, common themes related to the operationalisation of internationalisation were identified. They will be addressed in detail in the findings section. The findings indicate that there is a distinction in both focus and level of sophistication or articulation between the internationalisation strategies of research focused universities and more teaching focused institutions. Common themes include the rationalisation of international institutional links, the consolidation of internationalisation activities within the framework of a comprehensive strategic partnership, the focus on research as a driving force of international institutional links, excellence, as well as a renewed interest in Europe as a region necessitating attention. For a theme to be considered, it had to be raised by at least one interviewee across two institutions of a similar profile (i.e., broadly defined as teaching focused with an applied research agenda or research-intensive and research-led institutions). The theme had to capture something important about the data in relation to the research question while demonstrating some level of repetition across the data set, thus creating some level of patterned response (Braun & Clarke, 2006).

Sarantakos (1998) argues that qualitative analysis should occur “in waves, each wave following the previous one and providing additional information” (p.320). Interview transcripts were scrutinised several times to identify emerging themes. This approach has been confirmed by Braun and Clarke (2006) who recommend reading “through the entire data set at least once before you begin your coding, as ideas and identification of possible patterns will be shaped as you read through” (p.87). They suggest that the purpose of a thematic analysis, and its attraction for the researcher is based on its ability “to tell the complicated story of your data in a way which convinces the reader of the merit and validity of your analysis” (p.93). An inductive thematic analysis approach was utilised for analysing the transcripts to identify patterns of meaning across the dataset. These patterns were identified through a rigorous process of data familiarisation with all 24 interview transcripts, data coding, theme development and revision.

## Findings

It emerged from the data that research focused universities had different priorities than teaching-oriented universities when it came to internationalisation with a focus on Europe. The findings have been structured accordingly, based on broad institution type, to reflect commonalities between institutions occupying similar positions within the field.

### Strategic partnerships in teaching-focused institutions with a more applied research agenda

Eight participants (n = 8) in four institutions (Northern post-1992 university, Midlands post-1992 university, Northern Irish university and post-1992 Welsh university) that can be categorised as having a more teaching-focused mission were included, three of them developing from polytechnics under the UK’s previous binary system of higher education. The Further and Higher Education Act of 1992 unified the sector by allowing polytechnics to rename themselves as universities, although their subordinate position in the field quickly led to them being referred to as ‘post-92’ universities. The data extracted from the interviews shows that within these institutions, the priority is less focused on bilateral or consortia agreements with foreign universities, highlighting the importance of the research component as a driving force in forging international institutional linkages. While student exchange remains an important objective, the latter does not appear to be a sufficient rationale to invest significant human resources into developing comprehensive partnerships solely based on this dimension of internationalisation. Since status is associated with research, pursuing international institutional links based solely on student exchange, and therefore the teaching dimension, does not appear to warrant investing significant human resources. This has consequences for the internationalisation of higher education and global flows of research, that rely on the interconnectedness of universities, which appear to concern primarily universities with a minimum threshold of research intensity in a broad spectrum of fields and belonging to a subfield of elite research universities.

Within this sample of institutions, while collaboration with Europe was overwhelmingly viewed as desirable, the structure of this collaboration remained vague and was largely described in aspirational terms, with one participant characterising their institution’s approach to European collaboration as “enthusiastic” (Pro Vice-Chancellor, Northern post-1992 university), while also acknowledging the more applied research and teaching focus of their institution, in contrast to research-intensive institutions (Director, Northern post-1992 university).

Nonetheless, the delineation of research versus teaching tasks is not straightforward and one must be cautious about oversimplifying a university’s mission based on its history. The monopoly of research does not belong to established universities. Higher education institutions are dynamic institutions and organisational change is inevitable, and in time will lead to a challenge of the existing hierarchy of institutions in higher education systems that are vertically differentiated (e.g., Australia, Canada, the UK and the USA). Many ‘post-92’ universities excel in “pockets of international quality research”, and collaboration in these areas with European partners remains crucial:

As a post-92 in the UK, we have pockets of international quality research, but we don’t have the whole kind of breadth of international research that a Russell Group institution would, you know, Manchester, they will have had a much broader spectrum of international level research (Senior corporate executive, post-1992 Welsh university).

This view was reiterated by interviewees in all four institutions. Pockets of research excellence are crucial in driving the overall research capacity of these institutions. Excellence in research is a long-term project that needs time and resources to develop, and because universities are dynamic institutions, research excellence in one area can have spill-over effects into another related field. Nonetheless, it did not emerge from the interviews with participants in these universities that there were specific plans to remain connected in an institutionalised way (through international institutional links) to EU or non-EU universities.

Among these institutions, only one interviewee mentioned a potential move towards rationalising strategic partnerships, although there were no definite plans at the time of the interview in June 2018:

I think in terms of approach it will remain the same. I think what will happen is that we will probably move down a line of more an increase in the level of strategic partnerships, so what I would say is while at the moment we’ve got a number of strategic partnerships, we’ve then maybe also got a tail of other partnerships that cover various levels of interaction. I think we’ll probably look at how we might increase the number of high-level strategic partners that we’re working with, so rather than kind of, so there might be less volume of partnerships, but I think the in-depth relationships will probably change (Director, post-1992 Midlands university).

The findings indicate that the habitus of teaching focused institutions does not generate a disposition whereby the institution would act or think a certain way with regard to the need to instigate international institutional links in times of political turmoil and potential spill-over instability into the field of higher education. Institutional practices towards mitigating a potential impact of loss of access to European institutions and framework programmes that would prioritise autonomy from high-level politically negotiated frameworks for teaching and research collaboration (Erasmus+, Horizon Europe) and allow for greater security and independence from the political field were not considered essential, confirming the subordinate position of these institutions in the field. This rather cautious and embryonic approach towards international institutional links was not echoed by participants working in research-intensive universities.

### Strategizing the global space in research-intensive and research-led universities

Eight institutions (Northern Russell Group university, North East Russell Group university, South West Russell Group university, London Russell Group university, London specialised institution, Midlands research-led university; Scottish research-led university 1, Scottish research-led university 2) included in this study can be characterised as either research-intensive or research-led institutions, including one specialised university located in London (see Table 1). Four are members of the Russell Group (participants n = 8), two are former members of the now defunct 1994 Group of Universities (participants n = 4), one belongs to Midlands Innovation (participants n = 2), a group of eight self-proclaimed research-intensive universities in the latter geographically bound location, while one is currently non-aligned on the national scene (participants n = 2), in terms of membership purporting to represent the interests of specific type of university. Participants were unequivocal in highlighting a self-identified need to either create new or deepen existing partnerships with their European counterparts and beyond. Interestingly, the higher the scientific capital, as measured by research power (as measured by the Research Excellence Framework and success in securing EU funding under the Horizon 2020 scheme), the clearer an institution could articulate its internationalisation strategy in terms of international institutional links, and the more sophisticated its approach was. This seems to indicate that research focused institutions may be more inclined to seek international institutional linkages to maintain or reinforce their scientific capital.

Some participants (n = 3) in Russell Group universities highlighted that partnerships with European counterparts had not previously been prioritised, with Europe almost taken for “granted” (Vice Provost 1, London Russell Group university) compared to other regions with whom they had more structured linkages. Within the sample of four research-intensive universities that are members of the Russell Group, the most common approach mentioned by at least one participant from each institution to remedy this identified gap was to focus on European collaboration, through a small number of comprehensive strategic partnerships that were principally research driven. This emphasises the importance of the research dimension, because of the associated scientific capital, as a rationale for setting up comprehensive strategic partnerships, with a small number of identified “priority institutions” in Europe (Director, North East Russell Group university), invariably between three and six (Associate Vice President 1, Northern Russell Group university; Senior executive leader, South West Russell Group university; Senior Vice Principal, Scottish research-led university 2).

We’re a big university, so we have lots of people who are already working with Dr X in Copenhagen or Professor Y in Toulouse or wherever, which is fine, of course, bottom up, but we’re having sort of discussions about potential strategic partnerships with kind of, three or four European universities, which would be research driven really, in the same way that we have with, again, I suppose with universities such as Melbourne in Australia, you know. We’re talking with for instance the Technical University in Munich, at the moment, Copenhagen, the Free University of Amsterdam as well, but just to see if it’s worth sort of looking at things at an institutional level, which fits with our general strategy about trying to develop a number of strategic partners in different parts of the world (Associate Vice President 1, Northern Russell Group university).

The European aspect of engagement is linked to our global strategy and the global strategy is very simply explained, it is to move away from large numbers of thin involvements to a small number of deeper, thicker involvements and so our strategy for Europe is actually, in that sense, no different than our strategy for Australia or the US or South East Asia and so what we’re planning to do is to develop partnerships with a small number, maybe three or four institutions in Europe with whom we invest. The model is actually our relationship with [leading Australian research-intensive university], which you may have heard about already (Senior executive leader, South West Russell Group university).

Furthermore, it was clear that these institutionalised international links would provide an anchor for many dimensions of internationalisation indicating that institutional practices of this elite subfield of institutions were relatively sophisticated:

With [leading Australian research-intensive university], we have a multimillion-pound investment, roughly a million a year each, to set up PhDs, joint PhDs, we’ve got 22 on PhDs, we’ve got staff research projects, seed corn funding and we’re developing that and the institutions we’re working most closely with are Geneva, Lund and Copenhagen. None of those three are at the same level of investment as [leading Australian research-intensive university], although we’re looking to move one or more of them to that. Our next global relationship will be with [leading research-intensive Chinese university], so we aim to have maybe half a dozen in the world, and we’d like two or three of them to be in Europe (Senior executive leader, South West Russell Group university).

The focus on a “consolidated” (Dean, Midlands research-led university) number of core institution-wide European strategic partners was also a priority within all three multidisciplinary research-led institutions, with the notable exception of a specialised university located in London, the latter partly explained because of its specific mission and profile. Regardless of this arguably understandable exception participants in all three multidisciplinary research-led institutions highlighted the student mobility component, and the importance of prioritising partnerships that could cater for larger numbers of students, rather than “little collaborations” (Senior Vice Principal, Scottish research-led university 1) that dealt with “penny numbers of students” (Senior Vice Principal, Scottish research-led university 2).

Both bottom up and top down initiatives appear to have played a role in selecting institution-wide bilateral partnerships with a small group of European institutions, to “reduce transaction costs” (Dean, Midlands research-led university). While the senior executive team is ultimately responsible for approving these institution-wide partnerships, according to a majority of participants they tend to be rooted in pre-existing collaboration between researchers within a UK university and an EU or non-EU university. Because of the finite nature of prioritised institution-wide strategic partnerships, a single institution can only, in all good faith, have a limited number of such agreements. Therefore, top down oversight is necessary to curtail an overly enthusiastic number of position-takings that would dilute the purpose and diminish the impact of such agreements. In the evaluation or planning process, not all pre-existing collaborative partnerships can be elevated to the level of a comprehensive strategic partnership, and benefit from the status and resources such agreements are allocated. This tension was noticeable for specialised subjects not necessarily offered by a majority of institutions, such as Astrophysics, where cross-border partnerships are primarily led by academics in the discipline, and where such partnerships are inherently specific to a particular discipline and can be difficult to align with institution-wide priorities to reduce “transaction costs” linked to internationalisation activities. In such cases, subject-specific bilateral linkages remain crucial to researchers, who cannot rely on institution-wide partnerships to facilitate their collaboration with foreign researchers. Further research into individual academics’ perspectives on this issue is warranted but is not within the scope of this paper.

Within the specialised London research-led institution that did not appear, according to participants, to go down the route of selecting a core group of overseas universities to work with, both participants were keen to emphasise the bottom-up process driving collaborations. This was an interesting finding and an exception that could be explained by the specific mission and original *raison d’être* of that institution, and the fact that in many cases, its existing partners tend to share a similar mission and constitute a finite number of institutions.

Research universities have a distinct habitus which in large part determines the position-taking strategies they select, which clearly appears to indicate a predilection for a small number of key international institutional links with reputable, research focused institutions of a similar or higher level. Research universities’ habitus generates analogous preferences in terms of the identified need and selection of foreign partners. Ultimately, it is those institutions whose habitus most closely aligns with “the dominant culture and logic of practice of the field who possess the most symbolic capital relevant to it” (Watson, 2013, pp.415–416). In this sense, the dispositions of the habitus are “structuring structures” (Bourdieu, 1993, p.5) because of their capacity to generate practices adjusted to specific situations, in this case the uncertainty created by the Brexit negotiations. The research productivity of an institution is part and parcel of its scientific capital, through cutting-edge research published in high impact journals or through important scientific advances (e.g., the development of the Oxford AstraZeneca COVID-19 vaccine). This interbreeding between research universities is consistent with Bourdieu’s “general science of the economy of practices” (Bourdieu, 1993, p.8) which enables one to analyse all university practices (that originate in agents’ learned dispositions) as economic practices ultimately tailored towards maximising either material or symbolic profit.

According to participants in these institutions, favourite potential or existing (with a view to be strengthened) partnerships in Europe included almost invariably institutions occupying dominant positions in the field, such as the University of Amsterdam, the University of Copenhagen, Delft University of Technology, the Katholieke Universiteit Leuven (KU Leuven), the Ludwig Maximilian University of Munich, Lund University, Paris-Saclay University, Paris Sciences et Lettres University, Sorbonne University, the Technical University of Munich, Utrecht University and the Swiss Federal Institute of Technology in Zurich. These multidisciplinary research-intensive institutions also tend to feature in the top 100 of at least one if not all three of the most popular world university rankings (THE, QS, Shanghai). All but four (London Russell Group university, Scottish research-led university 1, Northern Russell Group university and North East Russell Group university) British partners could avail of such high positions in international rankings (as measured by featuring in the top 100 of at least one ranking) or in the breakdown of their research activities (in particular the London Russell Group and the Northern Russell Group institutions). A senior manager at a London Russell Group university confirmed this narrow focus on ‘excellence’ when selecting a foreign partner. The three partner institutions mentioned were the same institutions mentioned by their colleague, demonstrating the extent of consensus amongst the senior management team on the prioritisation of a small group of strategic partners, all in similar dominant positions within the field:

I think over the next 10 or 15 years what will develop is a small network, and I’m using the word network deliberately, of absolute topflight global universities, all of whom are very globally active, who all have links with the others. So increasingly, as we are identifying strategic partners, and we’re talking to them about their global engagement approach, the same partners crop up and often it’s about excellence works to excellence. So when we talk to Beida [Peking University] in Peking, PKU, we know they’re also in touch with the University of Toronto, they’re in touch with Yale, etc. So I think, over the next 10 or 15 years, rather than more focus on campuses, the top universities in the league tables, often a subset are really, really globally engaged, are already talking to each other. Perhaps not a total match, but it overlaps (Vice Provost 1, London Russell Group university).

I think we’re looking to have stronger relationships, rather than reducing, if you see what I mean, so we would never try to intervene to prevent a partnership developing between a collaborator, you know, at the PI level… but we might think institutionally of developing some stronger bilateral strategic relationships within the EU than we were thinking about before the Brexit issue came up. I think historically we thought, well, we were thinking about our bilateral relationships on a global scale, Peking, Yale, Toronto, whatever and we sort of took Europe as a given (Vice Provost 2, London Russell Group university).

Although there was some recognition of the role played by bottom up initiatives stemming from individual faculty members, the streamlining of international institutional links was confirmed by another senior manager in a Russell Group university located in the north of England, and while other links may survive, they will not be elevated to the strategic institution-wide level (Associate Vice President 2, Northern Russell Group university).

## Discussion

In UK universities, the international institutional links dimension of research universities has increasingly gravitated towards becoming a high-stake issue overseen by an institution’s senior management team, to the detriment of the diversity of partnerships individual academics may pursue. These institution-wide links become the canvas for many other dimensions of internationalisation, centralising and rationalising internationalisation activities under overarching comprehensive strategic partnerships, that become multidimensional (e.g., student exchange, international research collaboration, etc.) but also fewer in number, and closely aligned to institutional priorities set by senior leadership. The multidimensional focus of these links aligns itself with the concept of “comprehensive internationalisation” which is framed as an “organizing paradigm to think holistically about higher education internationalization” (Hudzik, 2011, p.5), to be understood here as the guiding paradigm applied to the logic of international institutional links that must demonstrate a “commitment confirmed through action, to infuse international and comparative perspectives throughout the teaching, research, and service missions of higher education. It shapes institutional ethos and values and touches the entire higher education enterprise” (Hudzik, 2011, p.6). It can also find some resonance in the European Universities Initiative, launched in 2017, which has led to the creation of transnational alliances of higher education institutions developing long-term structural and strategic cooperation in their core tasks of teaching, research and innovation, although unlike European Universities whose member institutions are required to originate from different parts of Europe, UK universities’ international institutional links with European counterparts have a tendency to focus on core Western countries, because of an overriding elitist imperative (Kleibert, 2023) due to the inherent requirements of the structuring of the field. Stability and continuity appear to guide the ‘partnership politics’ of institutions regardless of Brexit, an exogenous shock one might have anticipated to challenge the established habitus of those UK universities.

It is the senior executive level that evaluates, negotiates, monitors and renews such partnerships, with the support of academics, who were once the instigators of bilateral ties (Lane & Kinser, 2017). Individual choices and researcher-led partnerships that do not align with institutional strategic priorities will not benefit from the same level of support as those streamlined international institutional linkages, that can only be few in number. This displacement of the academic within the university is yet another consequence of changes in university governance (Shattock & Horvath, 2019) and growing “new managerialism” (Deem & Brehony, 2005) within universities, relentlessly removing academics from decision-making processes within the university and any potentially discordant voices that do not align with the senior executive’s strategic direction. No potential asymmetrical institution-wide partnerships were identified by senior university staff, indicating a careful curating of potential links to fit with the institution’s profile, implying a rationalisation of international institutional links from the top, competing for academic and scientific capital through their selection of international partners. This is consistent with Bourdieu’s (1993) structure of the field, which is determined by the relations between the positions agents occupy in the field, the latter being structured in hierarchy in the sense that agents occupy dominant and subordinate positions. Any change in a higher education institution’s position would lead to a change in the field’s structure, which is dynamic by nature. It is therefore crucial for universities to monitor and uphold their position in the field, and partnering with institutions that occupy a similar, or if possible higher position in the field, can be understood as part of that overall effort. An asymmetrical partnership could potentially disrupt the network of objective relations between positions that institutions occupy in the field, which could in turn have repercussions on the structure of the field. This would not be in the interest of those institutions occupying the dominant positions. It is this network of objective relations between positions that directs, like an irresistible current, the strategies which the occupants of the different positions implement to defend or improve their positions (i.e., their position-takings). Institutions are therefore implementing individually strategies such as institution-wide comprehensive strategic partnerships with other institutions occupying a dominant position to defend or improve their positions in relation to other occupants. These position-takings are inseparable from the objective position occupied by a higher education institution “as a result of their possession of a determinate quantity of specific capital” (Naidoo, 2004, p.459). The objective position of an institution therefore determines in a large part the “space of possibles” (Bourdieu, 1993, p.30).

It did not emerge from the data that teaching-focused institutions had a similar focus with regard to bilateral institutional linkages with Europe, which we argue demonstrates that such partnerships are first and foremost based on a shared research agenda or motivated primarily by research, which has implications in terms of the snowballing effect of research collaboration, paving the way for teaching and student mobility, leading to an enhanced and deeper partnership. The findings highlighted the already visible trend of those British universities that dominate, or perceive themselves as dominating the field, to seek partners they believe are occupying a similar dominant position abroad. Such a self-interested move is rooted in neo-liberal and New Public Management theories, while also revealing of the reputational “iron cage” (DiMaggio & Powell, 1983) that permeates decision-making within universities. It testifies to the constant struggle for control over the interests and resources specific to the field. This streamlining of relationships was articulated by participants in all four Russell Group universities part of the sample, and all other research-led institutions bar the specialised London institution. This elitist focus in Europe aligns with Kleibert who argues that “ties of excellence with reputed institutions in the EU” (2023, p.205) are a priority for elite UK universities. However, concepts such as ‘elite’ or ‘world class’ are nebulous and ill defined (Shattock, 2017), and the research capacity and academic capital of these universities is variable, as demonstrated by the existence of an unofficial “Golden Triangle” (Shattock, 2017, p.11) group suggesting further layers of prestige. The Russell Group’s membership is large in comparison to associations representing research universities in other countries (e.g., the Australian Group of Eight, China’s C9 League, the French Udice). Only its most research-intensive members are legitimate contenders as dominant players in a (sub)field of global research universities. Partnership aspirations of smaller Russell Group universities (North East Russell Group university; South West Russell Group university) and research-led universities, whose sense of imagining lead to identify themselves in large mainland European research powerhouses are manifestations of strategies to improve their position and can be referred to as “position-takings” (Bourdieu, 1993, p.35). In knowledge-based economies permeated by neoliberalism and new managerialism, universities are constantly implementing practices to improve or defend their objective position in relation to others they occupy in the field. Institution-wide partnerships with overseas universities are therefore a manifestation of this struggle for control of the interests and resources which are specific to the field of higher education, but they tend to be limited to institutions whose habitus so inclines those agents to seek such arrangements and have the required academic and scientific capital.

## Conclusion

The openness of higher education is therefore relative and constrained. Institutions, within their social field, occupy a specific position. Every position, even the dominant one depends on the other positions constituting the field. The senior university executives and academic managers involved in institution-wide internationalisation or strategic planning activities have contended that an institution’s position nationally must be buttressed or mirrored through partnerships with foreign universities that can help the institution maintain or improve its position in the field. This is particularly obvious in research universities’ position-takings through international institutional links, and less of a reality with more teaching focused institutions, because research is one of the driving forces cementing international institutional links. This is because status is increasingly linked to research activity and impact, hence international institutional links with a core research dimension are perceived to be more deserving in terms of investment of resources than those focusing predominantly on teaching activities. Failure to align an institution’s position-takings within the appropriate “space of possibles” (Bourdieu, 1993, p.30) is considered detrimental to the position of a higher education institution within the global field of higher education. Research-intensive universities align themselves with identified counterparts that share similar levels of academic and scientific capital. Because of the nature of international institutional links, the latter must have the approval of the senior executive, that verify the suitability of the partner to match the institution’s own profile and position within the field. While such an approach is rational, it risks alienating or isolating certain disciplines and leave them without institutional support. Internationalisation is a resource intensive set of activities and faculties will need the support of the institution when their needs differ from the centre. The findings demonstrate that there is a reducible “space of possibles” (ibid.) when looking at potential partners in Europe, and such findings may have similar (or exacerbated) outcomes in other regions of the world, in particular in the Global South, with research universities in the UK only partnering with an increasingly restricted number of institutions. The diversity of research partners and study abroad destinations are at risk of being substantially diminished and streamlined to an increasingly homogeneous subset of institutions, ring fencing international research collaboration and student mobility to a select few, most of which are located in the Global North. Institution-wide comprehensive strategic partnerships are set to become anchor frameworks for deeper collaboration and encompass many of the other dimensions of internationalisation that will find themselves entrapped under the rationale driving international institutional links with the practices of a subgroup of universities in the field, categorised here as research-intensive or research-led universities, oriented towards defending or improving their positions (i.e., through their position-takings).
